# Supplementary material for: Adipose Stromal Cell-Derived Secretome Attenuates Cisplatin-Induced Injury In Vitro Surpassing the Intricate Interplay between Proximal Tubular Epithelial Cells and Macrophages
Source: Cells. 2024 Jan 9;13(2):121. doi: 10.3390/cells13020121 (PMC10814170; doi:10.3390/cells13020121)

Supplementary Figure S5

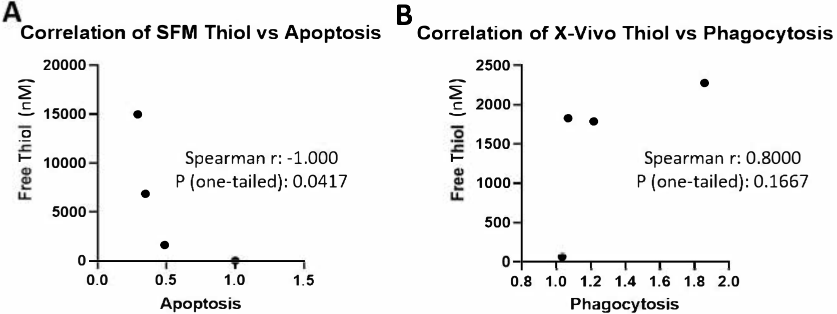

Supplement: Supplementary file 1 [file cells-13-00121-s001.zip › Supplementary Figure S5.pdf]
