# Supplementary material for: Adipose Stromal Cell-Derived Secretome Attenuates Cisplatin-Induced Injury In Vitro Surpassing the Intricate Interplay between Proximal Tubular Epithelial Cells and Macrophages
Source: Cells. 2024 Jan 9;13(2):121. doi: 10.3390/cells13020121 (PMC10814170; doi:10.3390/cells13020121)
Supplement: Supplementary file 1 [file cells-13-00121-s001.zip › Table S1.pdf]

**Supplementary Table S1. Primer sequences used for qPCR**

| Gene | Primer Sequence (5'-3') | |
|---|---|---|
| GADD45A | F | GCTCAACGTAATCCACATTC |
| | R | GAGATTAATCACTGGAACCC |
| CDKN1A | F | CAGCATGACAGATTTCTACC |
| | R | CAGGGTATGTACATGAGGAG |
| HMOX1 | F | CAACAAAGTGCAAGATTCTG |
| | R | TGCATTCACATGGCATAAAG |
| ATF3 | F | AGAAAGAGTCGGAGAAGC |
| | R | TGAAGGTTGAGCATGTATATC |
| GAPDH | F | TCCACTGGCGTCTTCACC |
| | R | GGCAGAGATGATGACCCTTTT |
