# Supplementary material for: Adipose Stromal Cell-Derived Secretome Attenuates Cisplatin-Induced Injury In Vitro Surpassing the Intricate Interplay between Proximal Tubular Epithelial Cells and Macrophages
Source: Cells. 2024 Jan 9;13(2):121. doi: 10.3390/cells13020121 (PMC10814170; doi:10.3390/cells13020121)
Supplement: Supplementary file 1 [file cells-13-00121-s001.zip › Table S2.pdf]

Supplementary Table S2. The concentrations of analytes in the co-culture supernatant measured using LUMINEX 200

			Analytes (pg/ml)																				
			HSP60	IL-1b	MMP-9	IP-10 (CXCL10)	IL-33	TIMP-1	IL-8 (CXCL8)	IL-10	IFN-b	IL-13	IL-1RA	Fractalkine (CX3CL1)	Rantes (CCL5)	GM-CSF	TNF-a	HGF	Arginase-1	M-CSF	Survivin (BIRC5)	VCAM-1	S100A8/A9	PDGF-BB
ciPTEC Monoculture	UT-CTRL	Mean	197	BDL	1	4	10	ADL	815	1	BDL	BDL	BDL	2	BDL	33	BDL	BDL	BDL	15	BDL	12	BDL	BDL
		SD	12	0	0	0	0	0	906	0	0	0	0	0	0	10	0	0	0	0	0	3	0	0
		N	3	3	3	3	3	3	3	3	3	3	3	3	3	3	3	3	3	3	3	3	3	3
	Cis-CTRL	Mean	156	BDL	4	4	11	65532	266	1	6	0	BDL	2	BDL	21	6	13	8	14	BDL	8	BDL	24
		SD	23	0	4	1	1	0	90	0	0	0	0	1	0	4	0	0	0	2	0	0	0	0
		N	3	3	3	3	3	3	3	3	3	3	3	3	3	3	3	3	3	3	3	3	3	3
ciPTEC-Macrophage Co-culture	UT-CTRL	Mean	2237	5	1577	22	25	ADL	2178	10	27	17	5633	8	5	115	55	146	21	ADL	307	31	13846	50
		SD	451	2	147	8	7	0	242	5	10	4	4894	3	1	39	22	80	9	0	70	10	2783	13
		N	3	3	3	3	3	3	3	3	3	3	3	3	3	3	3	3	3	3	3	3	3	3
	Cis-CTRL	Mean	1515	3	1212	15	15	ADL	2845	4	11	10	2654	5	3	61	27	97	BDL	ADL	216	19	11868	39
		SD	241	1	498	4	3	0	2163	1	5	4	2412	1	1	18	9	48	0	0	56	5	1644	14
		N	3	3	3	3	3	3	3	3	3	3	3	3	3	3	3	3	3	3	3	3	3	3
Macrophage Monoculture	UT-CTRL	Mean	2608	4	1425	21	22	28393	1813	8	22	14	6435	6	4	97	44	140	17	ADL	237	24	13483	45
		SD	1131	2	510	5	6	17227	1152	3	8	4	7546	2	1	28	14	71	6	0	43	6	2998	7
		N	3	3	3	3	3	3	3	3	3	3	3	3	3	3	3	3	3	3	3	3	3	3
	Cis-CTRL	Mean	1915	2	1587	17	14	70966	3358	4	9	8	2964	3	3	54	22	100	BDL	22	155	15	11947	35
		SD	418	0	256	4	1	52589	2321	1	3	2	2685	1	1	7	2	51	0	0	2	1	1196	4
		N	3	3	3	3	3	3	3	3	3	3	3	3	3	3	3	3	3	3	3	3	3	3
ciPTEC Monoculture	UT-CM	Mean	BDL	BDL	BDL	2	10	ADL	312	1	BDL	BDL	BDL	BDL	1	26	7	217	BDL	ADL	BDL	15	BDL	44
		SD	0	0	0	0	0	0	102	0	0	0	0	0	0	6	0	20	0	0	0	3	0	6
		N	3	3	3	3	3	3	3	3	3	3	3	3	3	3	3	3	3	3	3	3	3	3
	Cis-CM	Mean	BDL	BDL	BDL	3	10	ADL	312	1	BDL	BDL	BDL	BDL	1	20	7	193	BDL	ADL	BDL	12	BDL	48
		SD	0	0	0	0	0	0	107	0	0	0	0	0	0	5	0	22	0	0	0	2	0	0
		N	3	3	3	3	3	3	3	3	3	3	3	3	3	3	3	3	3	3	3	3	3	3
ciPTEC-Macrophage Co-culture	UT-CM	Mean	BDL	4	65	9	16	ADL	912	3	8	9	3719	3	2	47	24	197	BDL	ADL	245	20	5705	56
		SD	0	0	24	6	6	0	474	2	0	7	2834	0	1	24	17	81	0	0	0	6	1496	7
		N	3	3	3	3	3	3	3	3	3	3	3	3	3	3	3	3	3	3	3	3	3	3

	Cis-CM	Mean	BDL	BDL	68	5	11	ADL	975	2	BDL	4	2082	BDL	1	23	11	177	BDL	ADL	BDL	14	6259	56
		SD	0	0	18	1	0	0	318	1	0	0	1880	0	0	6	2	48	0	0	0	2	2200	10
		N	3	3	3	3	3	3	3	3	3	3	3	3	3	3	3	3	3	3	3	3	3	3
Macrophage Monoculture	UT-CM	Mean	95	BDL	63	6	12	ADL	635	2	BDL	7	3158	BDL	2	27	15	196	BDL	ADL	109	14	6333	52
		SD	0	0	20	2	1	0	315	1	0	0	3193	0	1	10	6	61	0	0	0	2	1100	5
		N	3	3	3	3	3	3	3	3	3	3	3	3	3	3	3	3	3	3	3	3	3	3
	Cis-CM	Mean	BDL	BDL	62	5	12	ADL	833	2	BDL	5	1839	BDL	2	21	13	182	BDL	ADL	BDL	10	6203	53
		SD	0	0	17	1	1	0	318	1	0	0	1437	0	0	3	2	39	0	0	0	1	542	7
		N	3	3	3	3	3	3	3	3	3	3	3	3	3	3	3	3	3	3	3	3	3	3
cpgiPTEC Medium	CTRL		BDL	BDL	BDL	BDL	9	BDL	BDL	0	BDL	BDL	BDL	BDL	N/A	BDL	BDL	BDL	N/A	ADL	BDL	BDL	BDL	BDL
	CM	Mean	BDL	BDL	2	6	10	ADL	48	1	BDL	BDL	BDL	BDL	1	20	8	96	BDL	ADL	N/A	10	BDL	38
		N (pool)	3	3	3	3	3	3	3	3	3	3	3	3	3	3	3	3	3	3	3	3	3	3
Macrophage Medium	CTRL		BDL	BDL	BDL	BDL	9	N/A	BDL	0	BDL	BDL	BDL	BDL	BDL	BDL	BDL	BDL	N/A	ADL	BDL	BDL	BDL	N/A
	CM	Mean	BDL	2	2	15	22	ADL	47	6	15	11	BDL	3	2	83	37	431	BDL	ADL	BDL	23	BDL	69
		N (pool)	3	3	3	3	3	3	3	3	3	3	3	3	3	3	3	3	3	3	3	3	3	3

*BDL: below detection limit; #ADL: above detection limit; N/A: unquantifiable due to beads gating/doublets.
