# Supplementary figures and images for: Adipose Stromal Cell-Derived Secretome Attenuates Cisplatin-Induced Injury In Vitro Surpassing the Intricate Interplay between Proximal Tubular Epithelial Cells and Macrophages
Source: Cells. 2024 Jan 9;13(2):121. doi: 10.3390/cells13020121 (PMC10814170; doi:10.3390/cells13020121)

# Supplementary Figure S1

**A**

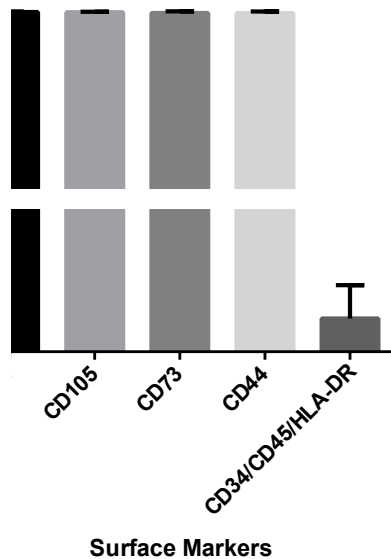

**B**

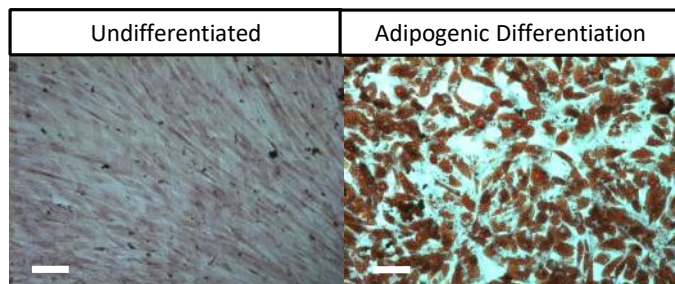

**C**

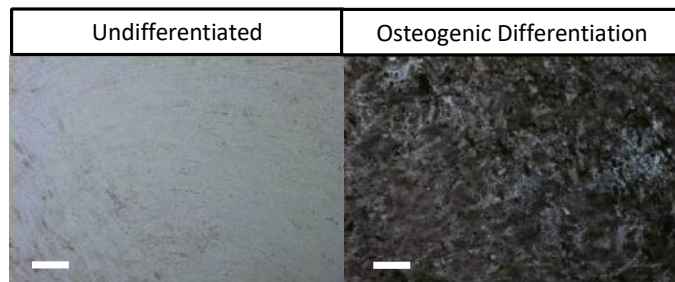

**D**

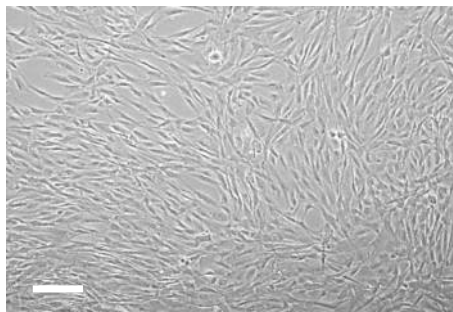

**E**

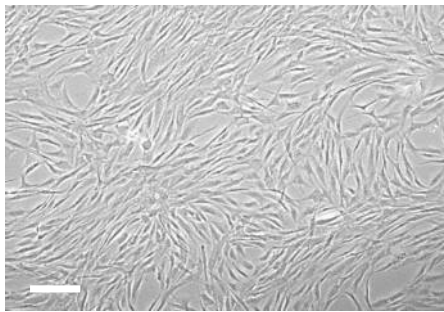

Supplement: Supplementary file 1 [file cells-13-00121-s001.zip › Supplementary Figure S1.pdf]

# Supplementary Figure S2

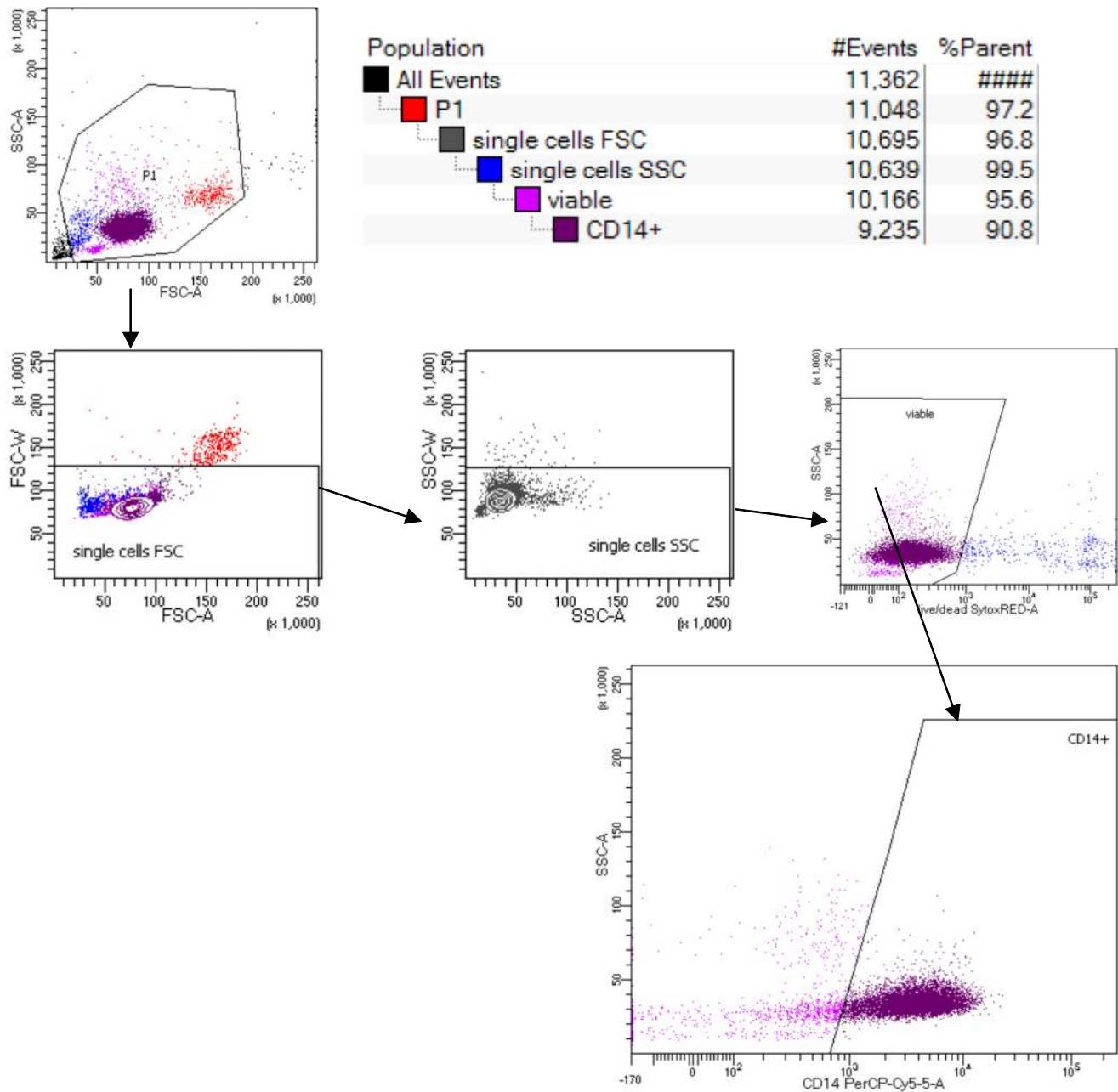

Supplement: Supplementary file 1 [file cells-13-00121-s001.zip › Supplementary Figure S2.pdf]

# Supplementary Figure S3

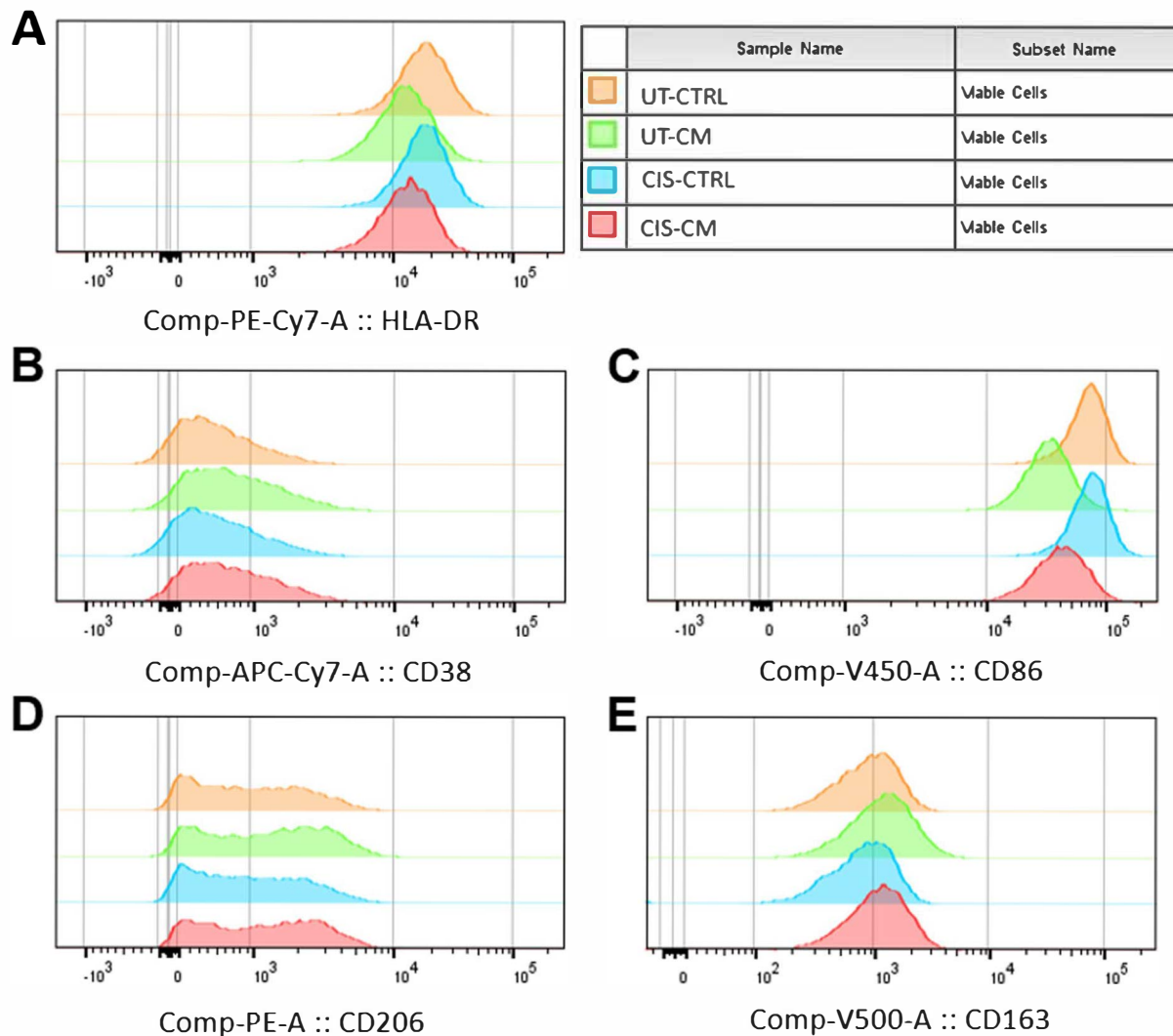

Supplement: Supplementary file 1 [file cells-13-00121-s001.zip › Supplementary Figure S3.pdf]

# Supplementary Figure S4

**A**

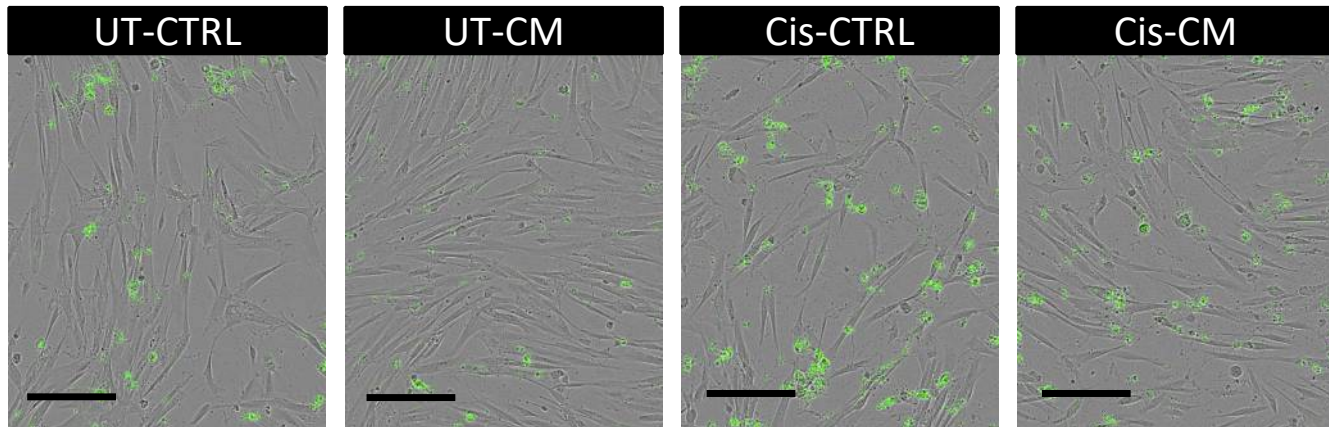

**B**

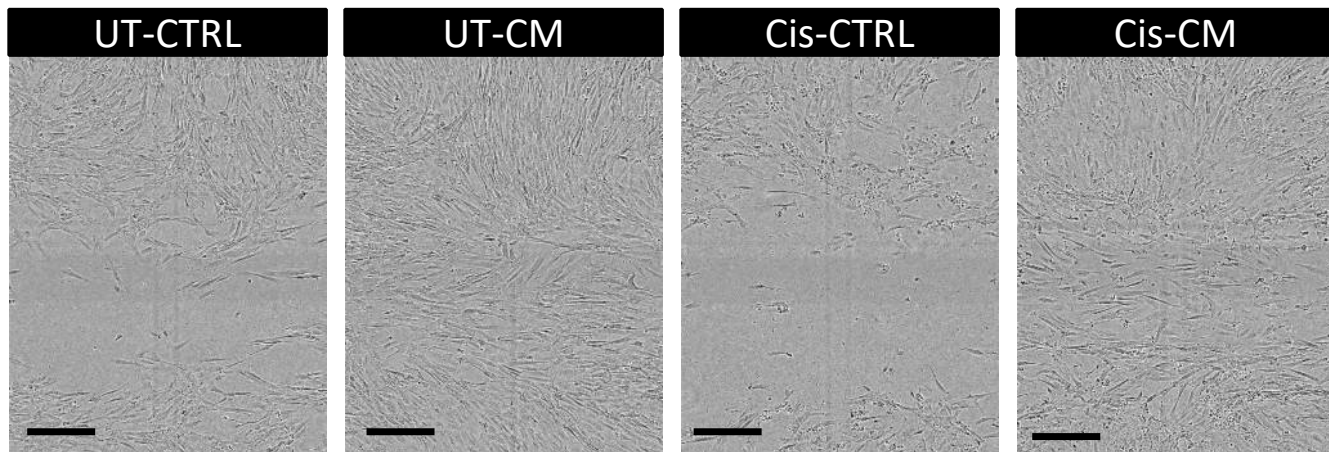

Supplement: Supplementary file 1 [file cells-13-00121-s001.zip › Supplementary Figure S4.pdf]

# Supplementary Figure S6

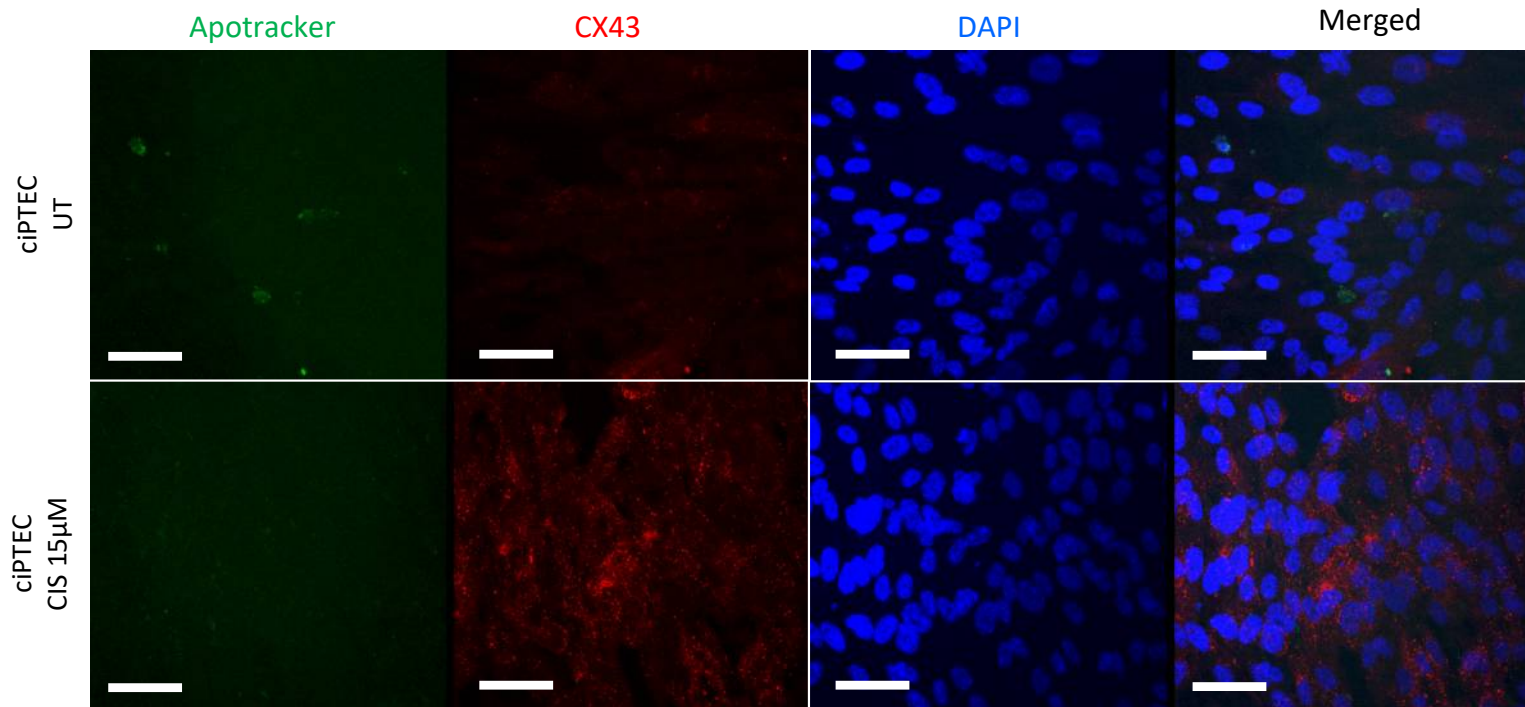

Supplement: Supplementary file 1 [file cells-13-00121-s001.zip › Supplementary Figure S6.pdf]

Supplementary Figure S7- part 1

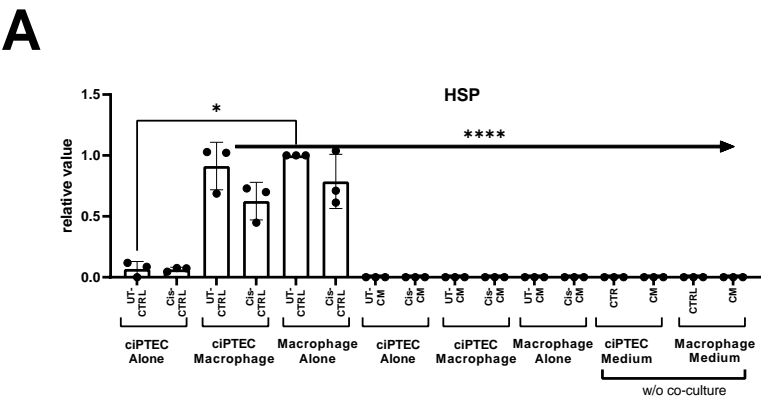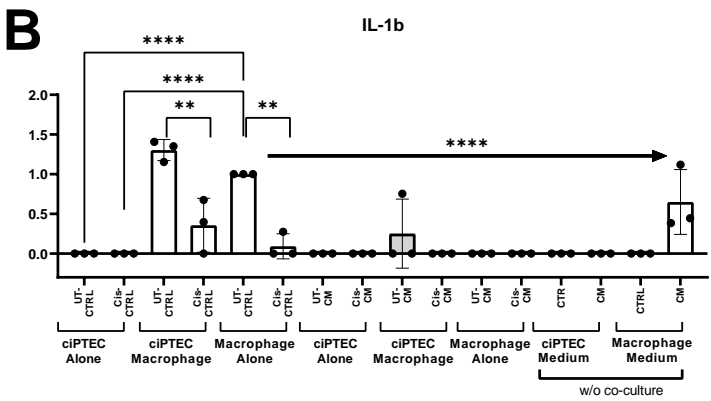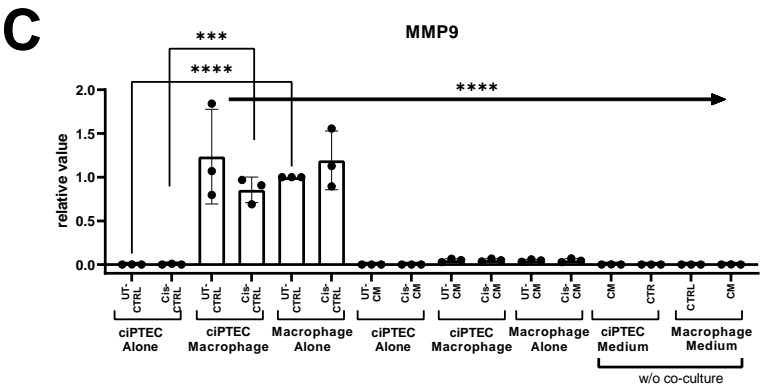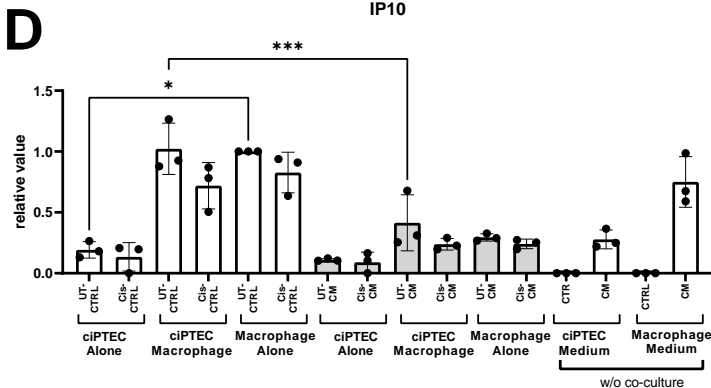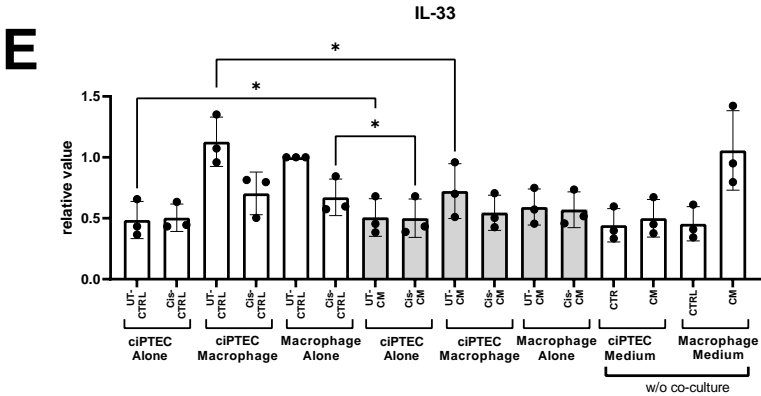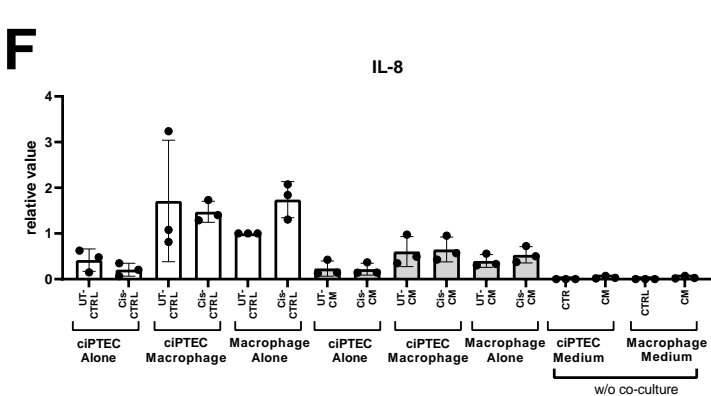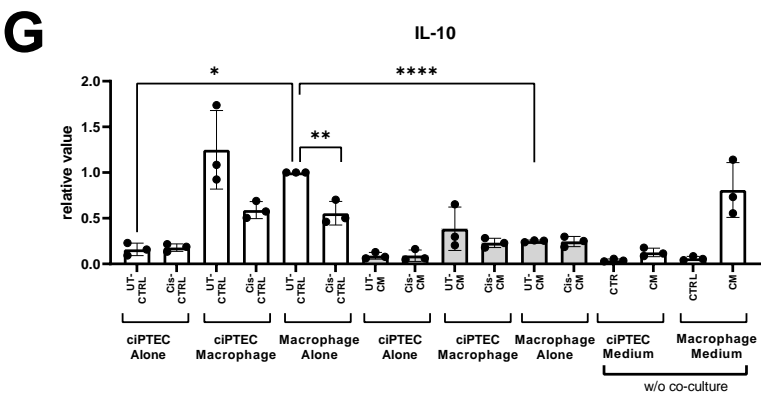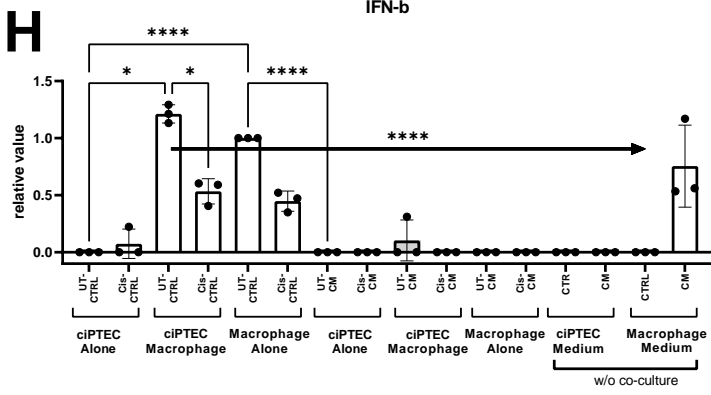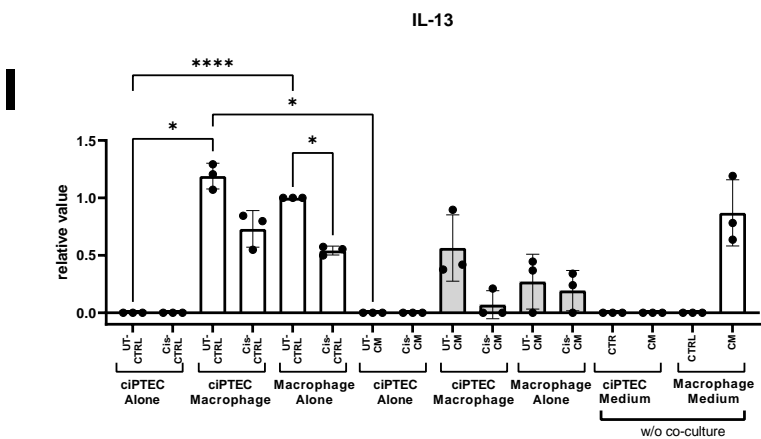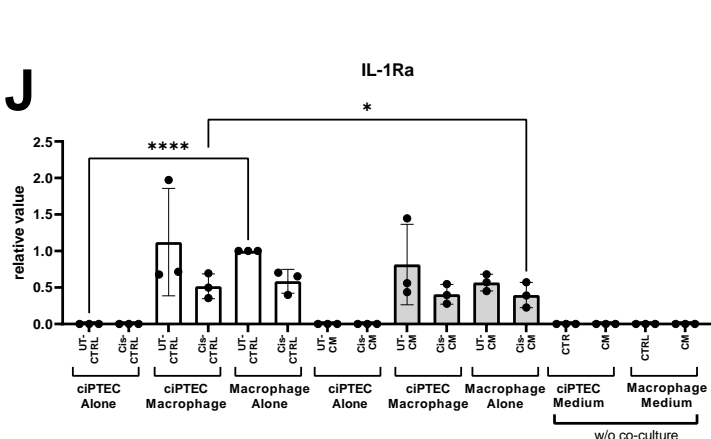

Supplement: Supplementary file 1 [file cells-13-00121-s001.zip › Supplementary Figure S7-part1.pdf]

## Supplementary Figure S7- part 2

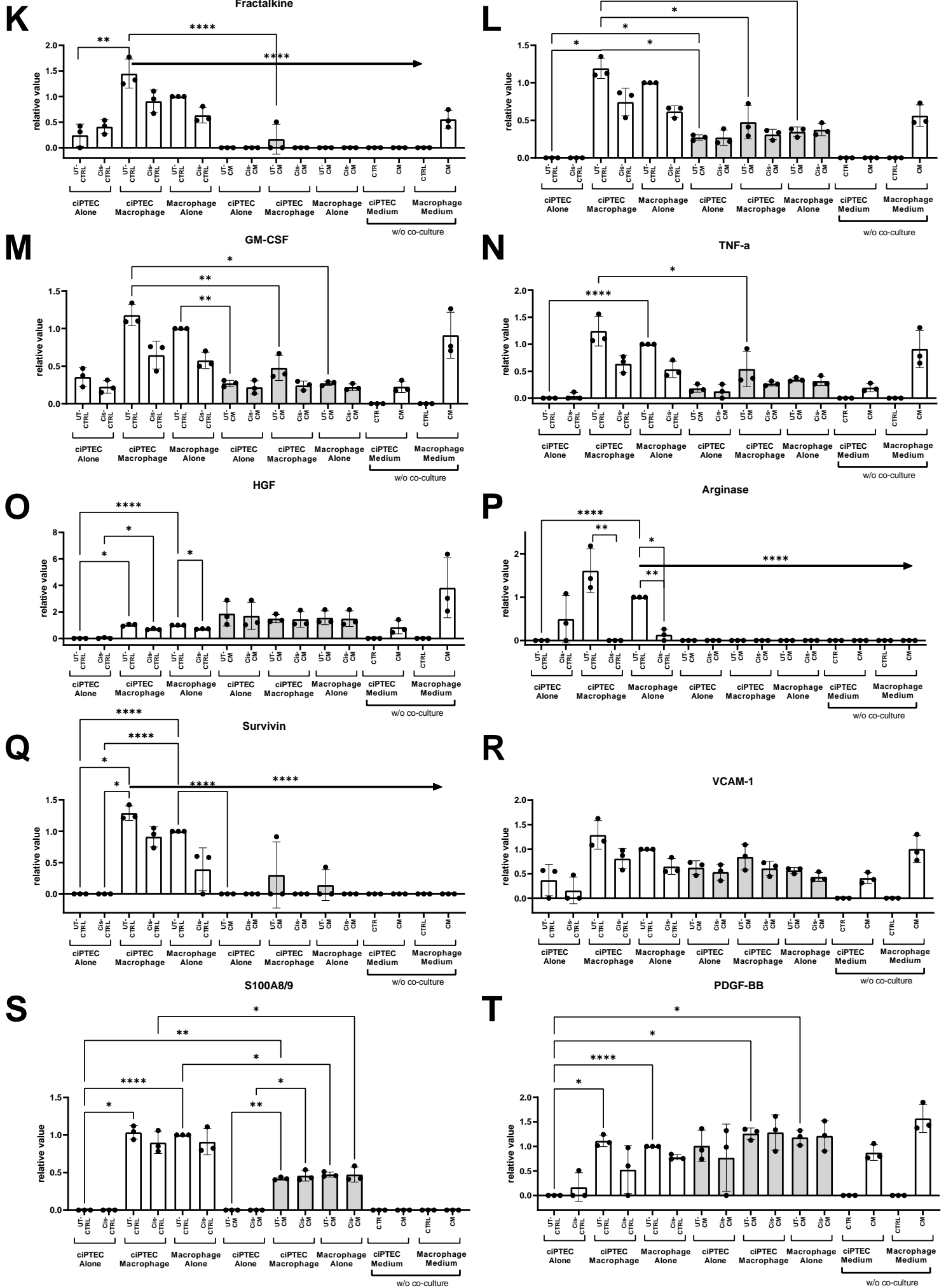

Supplement: Supplementary file 1 [file cells-13-00121-s001.zip › Supplementary Figure S7-part2.pdf]

# Supplementary Figure S8

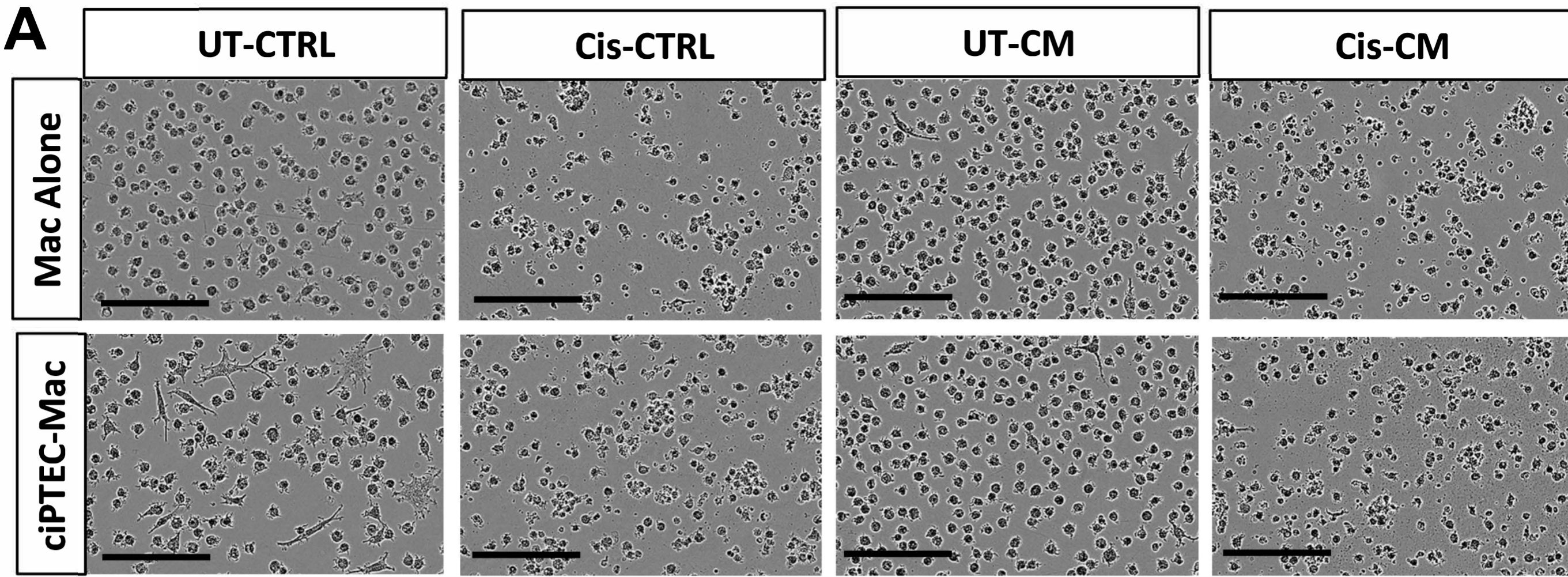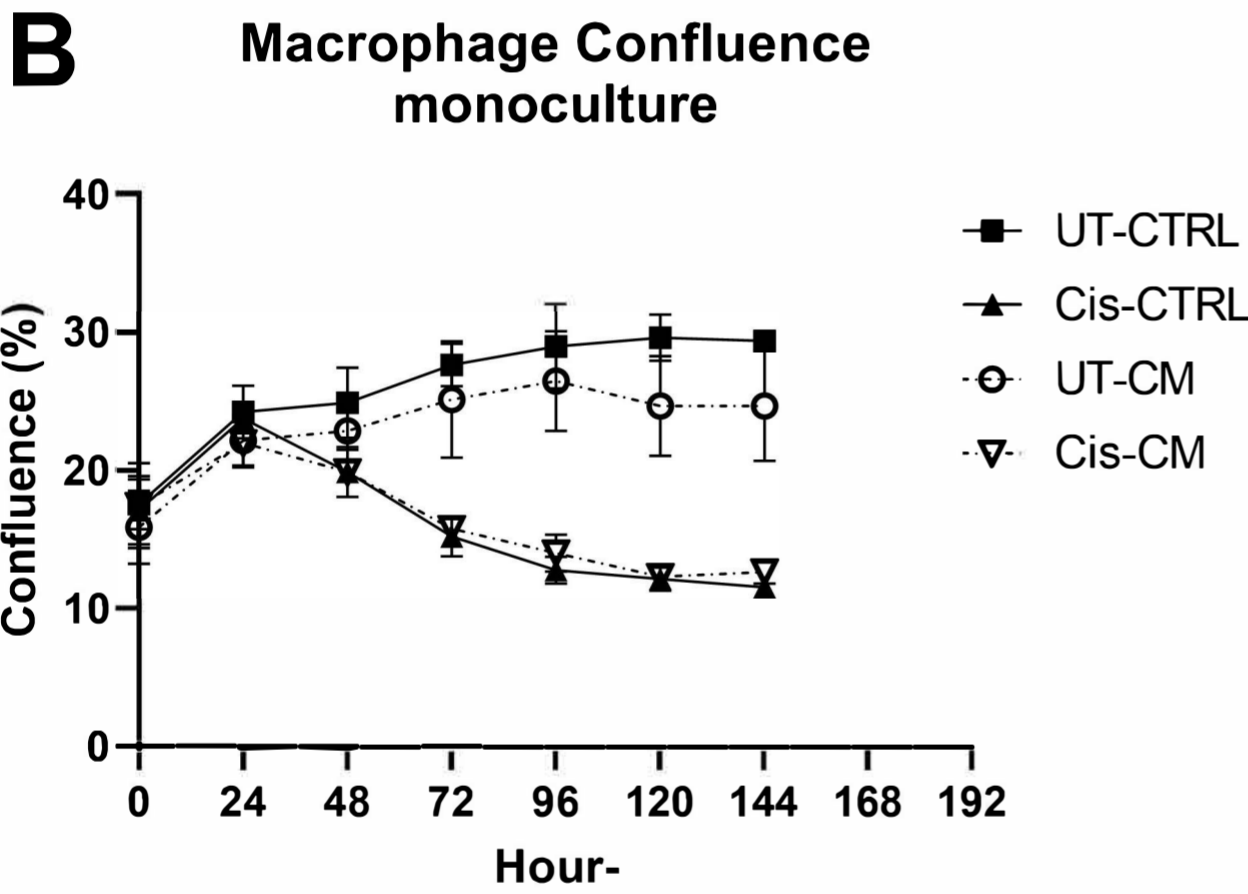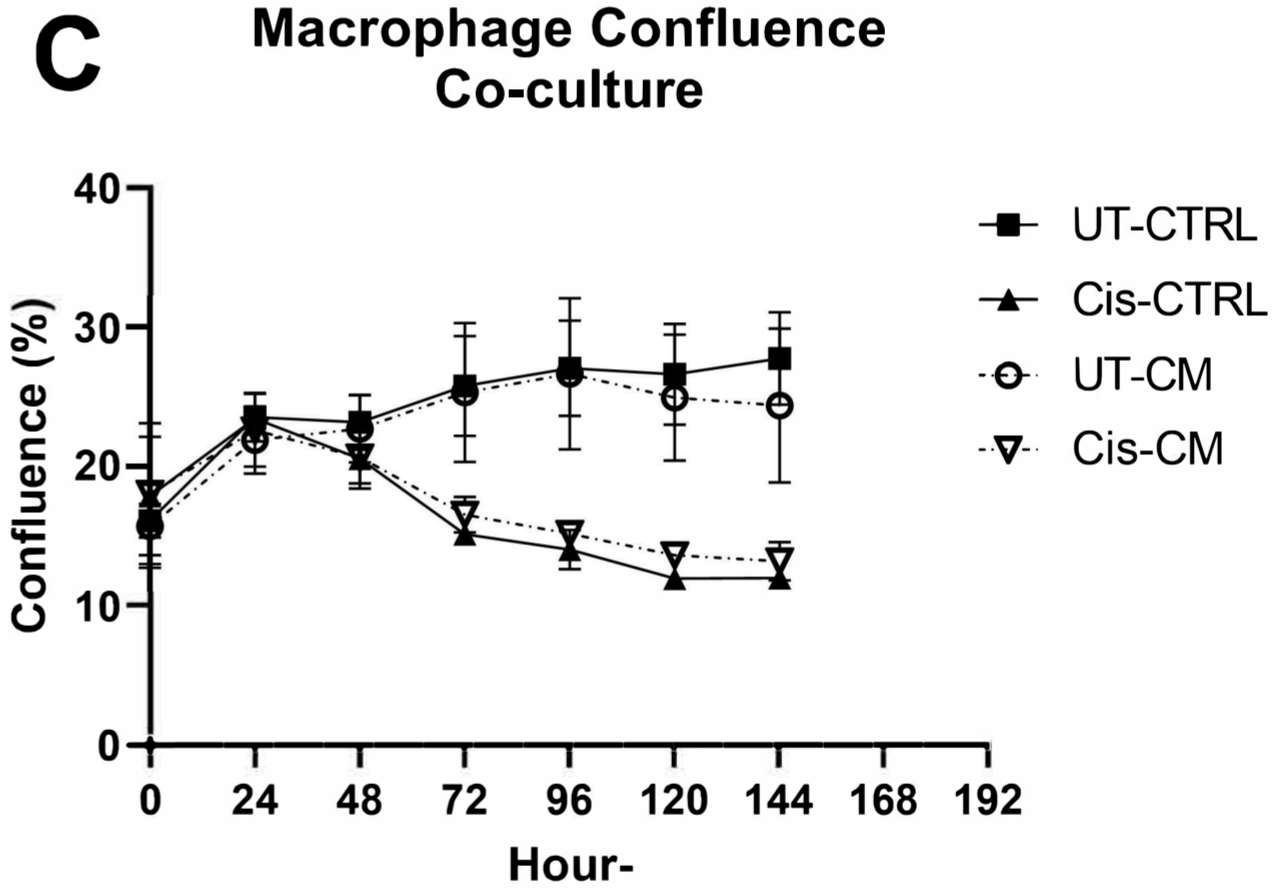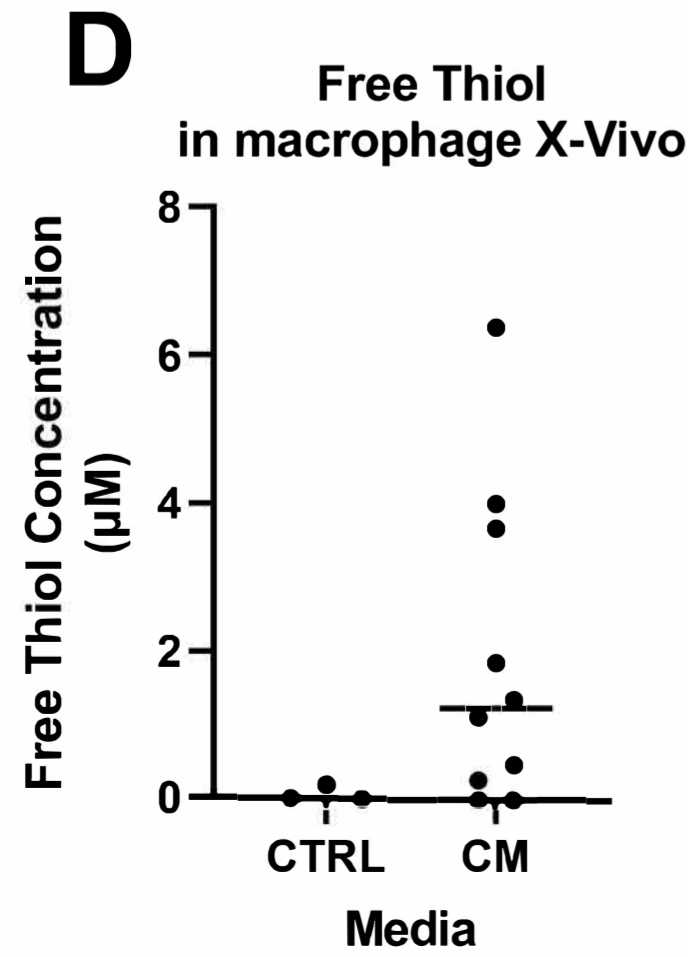

Supplement: Supplementary file 1 [file cells-13-00121-s001.zip › Supplementary Figure S8.pdf]
